# The impact of health resource enhancement and its spatiotemporal relationship with population health

**DOI:** 10.3389/fpubh.2022.1043184

**Published:** 2023-01-09

**Authors:** Leijie Qiu, Linsheng Yang, Hairong Li, Li Wang

**Affiliations:** ^1^Key Laboratory of Land Surface Pattern and Simulation, Institute of Geographical Sciences and Natural Resources Research, Chinese Academy of Sciences, Beijing, China; ^2^College of Resources and Environment, Chinese Academy of Sciences, Beijing, China

**Keywords:** population health, health resources, spatiotemporal, health technicians, regions

## Abstract

**Objective:**

This study investigated the impact of health resource enhancement on health and spatiotemporal variation characteristics from 2000 to 2010 at the county level.

**Methods:**

Multiscale Geographically Weighted Regression and curve fitting were used to explore the characteristics of spatiotemporal impact and divergence mechanism of health resource enhancement on population health.

**Results:**

From 2000 to 2010, China's population health continued to rise steadily, and health resource allocation improved. Population health demonstrated the significant spatial autocorrelation, and its spatial clustering patterns were relatively fixed. Health resource allocation was relatively equal. Health technicians per 1,000 persons had a significant positive effect on population health in 2000 and 2010. Meanwhile, its impact tends to be consistent across regions, and the impact scale has been continuously expanding. A quantitative relationship exists between population health and health resource inputs. When life expectancy ranged from 73.68 to 84.08 years, the death rate ranged from 6.27 to 9.00%, and the infant mortality rate ranged from 0.00 to 6.33%, investments in health resources, especially related to health technicians, were beneficial for population health.

**Conclusions:**

The government should improve the science and rationality of health resource planning. Planning meets regional realities by combining the impacts of economy and geography. The influence of health resources on population health depends on the overall allocation of health technicians. The number of health technicians needs to be further increased to improve the health resources' effective allocation between regions.

## 1. Introduction

Medical and health care is a basic guarantee of population health. Health resources are as the core of health care services (1), providing medical materials and supplies for population health. The relationship between health resources and population health has aroused the research interest of many scholars. They conducted a lot of studies related to the field. Many studies agree that the unequal distribution of health resources leads to varying levels of population health. Research has confirmed that health resources have a positive effect on population health. For example, Farahani et al. (2) examined the short- and long-term effects of changes in the number of physicians per capita on infant mortality. Regression results showed that an increase of one physician per 1,000 persons was associated with a 15% reduction in infant mortality within 5 years and a 45% reduction in the long term. Only by reducing interregional differences in health resource allocation and differences between social groups can we effectively improve population health. Based on a study of the health resource allocation in Ontario, Meyer (3) argued that the benefits of improving population health can be achieved by narrowing regional differences in health resource allocation. Michaels-Strasser et al. (4) noted that epidemic control cannot be achieved without a strong nursing and midwifery workforce at the frontlines of primary healthcare, based on a study of 13,387 nursing and midwifery students in the *Global Nursing Education Partnership Initiative* project. Lee et al. (5) found that lower OB-GYN availability in rural vs. urban counties explained most of the rural disadvantage in OB-GYN visit rates (83.8%), and much of the higher family physician (80.9%) and NP/PA (50.1%) visit rates. Bolstering the rural obstetrician–gynecologist workforce is important to mitigate maternal morbidity and mortality in the United States. Elma et al. (6) reported that physician shortage and maldistribution hinder patients' ability to access healthcare services in rural areas, which in turn contributes to a higher incidence of chronic diseases, injury, and mortality. Studies in China have shown that population health is affected by health resource distribution (7–9). Zhang (10) analyzed the dynamic impact of health resources on population health in Beijing and found that increased investments in health resources promoted population health and that the equality of health resource allocation between regions should be further improved. Gong and Chen (11) used geographically weighted regression to find that population health has a moderate positive correlation with health resources in southern China. For every 1 increased in the health resources index, population health index would increase by 0.503. At the same time, significant spatial heterogeneity was observed. Based on China's provincial panel data from 2009 to 2018, Yang et al. (12) measured the degree of coordination of population health and health resources using a coordination degree model and found an increasing trend. It rose from 0.379 in 2009 to 0.543 in 2018, with an average annual increase of 4.08%. The coordination degree values had clear regional differences and were more consistent with economic development.

Further review and analysis of relevant literature revealed that the existing studies had many limitations. In terms of the research scope, there have been many domestic and foreign studies on health resources and population health. Few studies have examined the correlations between health resources and population health. Among the causality studies, very few have summarized the influencing factors comprehensively and systematically. In terms of the selection of health resource indicators, most of the comprehensive studies that considered human, material, and financial inputs of health resources were conducted abroad. In contrast, the impact of health resources on population health in China is generally discussed only in terms of financial resources.

The impact of health resources on population health is a closed system circuit. It is influenced by multidimensional factors such as demographic change, economic development, and natural and social environments. The current study did not explore the correction variables (variables used for correction in addition to the independent and dependent variables in multifactor analysis). In terms of spatial scale, more studies have originated from within regions and rarely across regions. Intra-regional research was mostly focused on the eastern region, followed by the central region, with very few focused on the west. Second, the degree of spatiotemporal refinement is insufficient. Related studies that used provincial data have mainly been conducted on a national (13) or municipal data on a provincial (14, 15). There have been few studies on county-level data. In terms of temporal scope, static studies of cross-sectional data for a single year were predominant, while dynamic studies using spatial panel data for multiple years were less frequent.

Health is a variable state, and the spatiotemporal distribution relations between health resources and population health must also change over time. Therefore, it is difficult to grasp the spatiotemporal evolution in health resources and population health from a holistic perspective using data from a single year. In terms of research methods in population health and health resources, there has been a gradual shift from solely qualitative to a combination of quantitative and qualitative researches, mainly based on mathematical and statistical analyses. For example, related studies have used data envelopment analysis (16), logistic regression analysis (17), fixed-effects model (14), regression inequality index method (18), among others, to quantitatively analyze the relationship between health resources and population health. However, few studies have used geospatial analysis to perform a more detailed analysis of regional differences.

In the spatial analysis, empirical studies have demonstrated significant spatial heterogeneity in terms of the impact mechanism of population health (1, 19). Spatial methods such as ordinary lease square regression model (OLS) and geographically weighted regression (GWR) have been applied in health service accessibility (20) and health resource allocation (21). But specific studies using Multiscale geographically weighted regression (MGWR) to take into account local spatial heterogeneity have not yet emerged. GWR can address spatial heterogeneity problems that cannot be handled by traditional linear regression models to a certain extent. However, it ignores differences in the scales of different influencing factors' spatial heterogeneity, which leads to a large estimation bias. MGWR provides spatial impact scales of different variables by allowing different bandwidths for each variable, which addresses the shortcomings of GWR and generates more credible estimation results. Meanwhile, although semi-parametric geographically weighted regression (SGWR) can deal with global and local scale problems to some extent, it can only classify the influence scale of different variables into global and local categories and cannot be further subdivided (22). In contrast, the MGWR provides a substantial response to this deficiency (23, 24). The MGWR allows the range of data intervention (bandwidth) to vary over the entire parameter surface. It also allows the association between different independent and dependent variables to be inconsistent in scale. That is, the variables are allowed to be related to the dependent variable at their optimal bandwidths (25). Thus, the regression results of the MGWR are more reliable.

In summary, a study of the relationship between health resources and population health across years based on multidimensional influencing factors will contribute to scholarly literature. Counties are the basic spatial units and bearers of economic development and resource transfers. Cross-regional research on county health resources and population health is of practical significance. Geospatial analysis is an objective and scientific method for analyzing regional differences in the relationship between health resources and population health. Therefore, it is necessary to analyze the impact of health resource enhancement and its spatiotemporal relationship with population health at the county level. This study used MGWR method to analyze related data at the county scale across seven geographic regions from 2000 to 2010. Further, factor analysis was performed with multidimensional indicators. We believe that this study can provide a scientific basis for the optimal allocation of health resources and effective improvement of population health in China. It can also benefit the development of health geography.

## 2. Data and methods

### 2.1. Variable selection

Through a literature review, we retrieved research articles related to population health, the impact of health resource enhancement on health change, and the factors influencing population health from 2000 to 2022. The main research themes were population, environment, economy, population health, healthcare, education, housing, and town development. Combining expert opinions, high-frequency keywords—death rate (26), life expectancy (27), infant mortality rate (28), minimum temperature (29), normalized difference vegetation index (NDVI) (30), gross domestic product (GDP) per capita (31), urban and rural savings deposit balance (18), health facilities per 1,000 persons (32), health technicians per 1,000 persons (33), hospital beds per 1,000 persons (34), illiterate population aged 15 years and above (35), housing floor area per capita (36), and urbanization rate (37)—were used as research variables in this study.

According to the World Health Organization, reducing mortality, especially stillbirth and neonatal mortality, is a top public health priority. Internationally, death rate, infant mortality, and life expectancy are core indicators of a country's population health. The *Health China 2030 Planning Outline* stated that between 2015 and 2030, life expectancy will increase from 76.34 to 79.00 years and the infant mortality rate will decrease from 8.10 to 5.00%. Therefore, we used death rate, infant mortality rate, and life expectancy as the measures of population health. Factors that determine health are very complex and include natural environmental, economic, educational, and healthcare factors. Under certain environmental, economic, and educational conditions, changes in health resource inputs can lead to optimal health outcomes. We focused on the impact of health resource enhancement on population health. The utilization of health resources per 1,000 persons is not only an important indicator for evaluating the availability of health care services but also an important basis for measuring the fairness of health resource allocation. This study used health facilities per 1,000 persons, health technicians per 1,000 persons, and hospital beds per 1,000 persons as indicators of health resource allocation. From 2000 to 2010, China attached great importance to investment in county health resources. However, there is a need to better understand whether the increase in health resources at the county scale is conducive to improving population health and regional impact differences.

### 2.2. Data

We conducted an empirical analysis using publicly available government data. This study mainly covered data related to 2,856 counties across 31 provinces nationwide from 2000 to 2010. The basic data mainly included health data, health resource data, and other socioeconomic data.

#### 2.2.1. Health data

Health data were mainly derived from the fifth and sixth censuses of the 31 provinces, prefecture-level cities, and selected counties. The death rate was calculated as the ratio of total mortality to the total population from all age groups, represented as thousandths (%). Infant mortality was defined as the ratio of mortality among infants aged < 1 year (%). Life expectancy was calculated by current life table method. The current life table method uses a cross-sectional mortality rate in the current year of the study to prepare life tables. It assumes that future expected mortality is consistent with the current year of the study, and 1 year of data is required to prepare the life tables. Its formula for calculating life expectancy is illustrated in Table 1.

**Table 1:** Abbreviated life table for one region in China.

**x**	**P_x_**	**D_x_**	**m_x_**	**q_x_**	**l_x_**	**d_x_**	**L_x_**	**T_x_**	**e_x_**
0~	50,138	1,930	0.038	0.038	100,000.000	3,849.376	98,075.312	6,672,842.574	66.728
1~	198,325	1,024	0.005	0.020	96,150.624	1,965.499	380,671.499	6,574,767.262	68.380
5~	274,213	423	0.002	0.008	94,185.125	723.657	469,116.482	6,194,095.763	65.765
10~	293,296	328	0.001	0.006	93,461.468	521.144	466,004.478	5,724,979.281	61.255
80~	9,260	861	0.093	0.377	30,665.540	11,567.592	124,408.718	308,378.615	10.056
85~	4,383	455	0.104	1.000	19,097.947	19,097.947	183,969.897	183,969.897	9.633

From left to right, x represents age; D_x_ and P_x_ are, respectively, the number of deaths and the alive population at x year; M_x_ represents the probability of dying at age x; q_x_ represents the probability of death; l_x_ represents the number of people alive at that age; d_x_ represents the number of deaths in the interval (x, x + i) for persons alive at age; L_x_ represents the number of surviving person-years in n years for those who are still alive at age x; T_x_ represents the total number of person-years lived by the cohort from age x until all members of the cohort have died; e_x_ represents life expectancy.

#### 2.2.2. Health resource data

Health resource data were obtained from the 1999–2011 *China Health Statistical Yearbook, Statistical Bulletin on Health* and *Health Care Development, National Economic and Social Development Bulletin*, provincial *government work reports*, local and county *health statistical yearbooks*, and statistical websites. Among them, health technicians per 1,000 persons were calculated as the ratio of total health technicians at year-end to the resident population at year-end, multiplied by 1,000. The unit is represented by a person. Health facilities per 1,000 persons were the ratio of total health facilities at year-end to resident population at year-end, multiplied by 1,000. This unit is represented as an institution. Hospital beds per 1,000 persons were the ratio of total beds in health facilities at year-end to the resident population at year-end, multiplied by 1,000. The unit was represented as a bed.

#### 2.2.3. Socioeconomic data

The socioeconomic data indicators mainly were related to demographic statistics, administrative divisions, land use, climate, basic geographic information, GDP, education, real estate, urban development, and savings deposit. “Illiterate population aged 15 years and above” were obtained from the fifth and sixth censuses across the 31 provinces, prefecture-level cities, and selected counties. At the same time it was also obtained from the 1999–2011 *China Statistical Yearbook, Population and Employment Statistical Yearbook, China Education Statistical Yearbook*, statistical websites, and yearbooks of the provinces, cities, and counties. “Normalized difference vegetation index,” administrative division and basic geographic information data at 1:1,000,000 were obtained from the Resource and Environment Science and Data Center of the Chinese Academy of Sciences. “Minimum temperature” was obtained from the National Meteorological Administration and the Ecological Science Data Center of the Chinese Academy of Sciences. “GDP per capita” and “urban and rural residents savings deposit balance” were obtained from the 1999–2011 *China Statistical Yearbook, China Financial Statistics Yearbook*, and *the statistical yearbooks* of provinces and municipalities. “Housing floor area per capita” and “urbanization” were obtained from the 1999–2011 *China Statistical Yearbook*, C*hina Real Estate Statistical Yearbook, China Construction Industry Yearbook, China Urban Statistical Yearbook*, the fifth and sixth population census information of each province, provincial and prefecture-level city statistical yearbooks, construction statistical yearbooks, and real estate statistical yearbooks.

The change rate of all indicators used 2010 minus 2000 data. Since the national administrative region have changed considerably from 2000 to 2010, all of the data above were based on and corrected for the administrative region in 2013. In addition, to eliminate the effect of price indices, the cost category data were adjusted using a GDP deflator (2017 = 1). In the preliminary data processing, we performed data integration and calibration, numerical statistics and spatial data matching, as well as projection transformation and resampling. In addition, when building the database, every two entry clerks performed data cross-checking and multiple error checking to ensure data authenticity. For variables' summary statistics, see Table 2 for more details.

**Table 2:** Variable definitions and summary statistics.

**Variable names**	**Abbr**.	**Units**	**Mean**	**SD**	**Min**	**Median**	**Max**
**In 2000**							
Life expectancy	Life	Age	72.30	4.68	41.25	73.18	84.08
Death rate	Death	%	6.16	1.23	0.89	6.14	11.04
Infant mortality rate	IMR	%	23.72	17.85	0.57	19.45	110.59
Minimum temperature	TEMP	°C	−3.59	8.84	−32.37	−2.30	17.59
Housing floor area per capita	Housing	m^2^	21.91	6.15	4.76	21.05	52.40
Urban and rural residents savings deposit balance	Saving	Yuan	128,726.40	124,631.50	101.00	97,673.00	982,000.00
Urbanization	Urban	%	36.48	30.45	0.00	24.27	100.00
Illiterate population aged 15 years and above	Unedu	Person	136,238.20	173,427.50	1,170.00	48,215.00	820,685.00
Normalized difference vegetation index	NDVI	Index	0.64	0.15	0.07	0.70	0.84
GDP per capita	GDP	Yuan	593.56	468.62	62.40	461.46	4749.95
Health facilities per 1,000 persons	Hospital	Institution	0.20	0.24	0.01	0.11	2.91
Health technicians per 1,000 persons	Person	Person	3.78	3.00	0.44	2.97	24.78
Hospital beds per 1,000 persons	Bed	Bed	2.06	1.19	0.41	1.76	7.99
**In 2010**							
Life expectancy	Life	Age	77.97	3.33	60.12	78.05	87.97
Death rate	Death	%	5.92	1.34	0.78	5.92	11.16
Infant mortality rate	IMR	%	5.10	6.06	0.10	3.16	49.07
Minimum temperature	TEMP	°C	−3.43	9.24	−34.94	−1.17	18.70
Housing floor area per capita	Housing	m^2^	30.12	7.34	11.89	29.77	57.66
Urban and rural residents savings deposit balance	Saving	Yuan	566,926.00	539,698.40	1,043.00	440,320.00	5,970,400.00
Urbanization	Urban	%	45.51	21.27	6.11	40.02	100.00
Illiterate population aged 15 years and above	Unedu	Person	78,252.79	114,788.70	453.00	24,529.00	718,634.00
Normalized difference vegetation index	NDVI	Index	0.70	0.16	0.08	0.75	0.89
GDP per capita	GDP	Yuan	26,576.17	22,458.98	2,160.20	19,973.18	192,156.40
Health facilities per 1,000 persons	Hospital	Institution	0.57	0.82	0.01	0.28	8.97
Health technicians per 1,000 persons	Person	Person	4.87	3.62	0.51	4.03	31.86
Hospital beds per 1,000 persons	Bed	Bed	2.86	1.64	0.33	2.48	12.90

### 2.3. Methods

#### 2.3.1. Multiscale geographically weighted regression

MGWR is a software application developed by Professor Stewart Fotheringham (22) and calibrates GWR. It runs on both Microsoft Windows and MacOS. MGWR breaks the assumption that all models are on the same spatial scale and explores the spatial relationships between various types of variables at different spatial scales.

MGWR is calculated as follows:


$$\begin{array}{l} y_{i_{}}=\beta_{bw0}\left(u_{i},v_{i}\right)+\overset{m}{\underset{k=1}{\sum }}\beta_{bwk}\left(u_{i},v_{i}\right)X_{ik}+\varepsilon_{i} \end{array}$$

$$\begin{array}{l} \varepsilon_{i}\tilde{\;}N\left(0,\sigma^{2}I\right),i=1,2,...,n. \end{array}$$

In this equation, *y*_*i*_ is the response variable at spatial location i,*X*_*ik*_(*k* = 1, 2, ...*m*) is the explanatory variable at spatial location i, (*u*_*i*_, *v*_*i*_) is the coordinate of point i in the regression analysis,β_*bw*0_(*u*_*i*_, *v*_*i*_) is intercept term of the regression relationship, ε_*i*_ is a mutually independent random error term, β_*bwk*_(*u*_*i*_, *v*_*i*_) is the regression analysis coefficient. β_*bwk*_(*u*_*i*_, *v*_*i*_) is a continuous function of spatial location (*u*_*i*_, *v*_*i*_) and bandwidth used by the regression coefficient of the jth variable. β_*bwk*_(*u*_*i*_, *v*_*i*_) is obtained based on local regression and has specificity. That is, different regression coefficients can be calibrated to obtain different bandwidths. In this paper, we used Gauss (2.12) and akaike information criterion, corrected (AICc) criterion (2.13) as the kernel function and bandwidth selection criterion. MGWR can be viewed as a generalized additive model (GAM). β_*bwk*_*X*_*k*_ in the MGWR model is defined as the kth additive term *f*_*k*_ in the GAM. Therefore, the MGWR model in GAM form can be written as:


$$\begin{array}{l} y=\overset{m}{\underset{k=0}{\sum }}f_{k}+\varepsilon \end{array}$$

The MGWR is calibrated using the back-fitting method (maximize logarithmic likelihood) (Fotheringham, 2017). The estimated values of GWR model are chosen as its parameters' initial estimate. After convergence, the value of the differences between successive iterations was sufficiently small for the back-fitting iteration to terminate. We selected *SOC*_*f*_ as the termination criterion. The variation in the GWR smoothness can be expressed as follows:


$$\begin{array}{l} {SOC}_{f}=\sqrt{\frac{\sum_{k=1}^{p}\frac{\sum_{i=1}^{n}{\left({\overset{\hat{\;}}{f}}_{ik}^{new}-{\overset{\hat{\;}}{f}}_{ik}^{old}\right)}^{2}}{n}}{\sum_{i=1}^{n}{\left(\sum_{k=1}^{p}{\overset{\hat{\;}}{f}}_{ik}^{new}\right)}^{2}}} \end{array}$$

#### 2.3.2. Global and local Morans'*I*

Morans'*I* describes the association degree of attribute values of units spatially proximity or adjacent. It is extensively used to test the spatial autocorrelation based on feature locations and attribute values (38). The study employs global and local Morans'*I* to explore the clustering effect of population health. Global Morans'*I* examines spatial clustering of the whole spatial sequence. It is used to analyze whether there is spatial autocorrelation.

The calculation for global Morans'*I* is as following:


$$I=\frac{\sum_{i=1}^{n}\sum_{j=1}^{n}w_{ij}\left(x_{i}-\bar{x}\right)\left(x_{j}-\bar{x}\right)}{\sum_{i=1}^{n}{\left(x_{i}-\bar{x}\right)}^{2}}$$

Where,*x*_*i*_is population health in the region *i*, $\bar{x}$ is the average value, *n* is the number of the regions, *w*_*ij*_ is the element of spatial weight matrix (*i, j*), it is used to measure the distance between regions *i* and *j*, $\sum_{i=1}^{n}\sum_{j=1}^{n}w_{ij}$ is the sum of all spatial weights.

When global Morans'*I* appears to be autocorrelated, we proceed to local Morans'*I*. Local Morans'*I* is used to detect outliers or spatial clustering around a region *i*. It digs deeper into the correlation of characteristic values between specific spatial and adjacent spatial units (39).

The calculation for local Morans'*I* is as following:


$$\begin{array}{ll} I_{i}=\frac{\left(x_{i}-\bar{x}\right)}{s^{2}}\sum_{j=1}^{n}w_{ij}\left(x_{j}-\bar{x}\right) & \\ & s^{2}=\frac{\sum_{i=1}^{n}{\left(x_{i}-\bar{x}\right)}^{2}}{n} \end{array}$$

#### 2.3.3. Gini coefficient

This study used the Gini coefficient to measure regional differences in health resource allocation. The Gini coefficient can be understood as the relative degree of deviation of the average gap in health resource allocation (according to the population distribution) relative to the overall expectation.

The Lorenz curve can reflect the distribution share of resources among the population more intuitively and is often used to quantify the fairness and equality of the allocation of public service facilities. The Gini coefficient reflects the extent to which the Lorenz curve deviates from the line of absolute equity (40). Based on the Lorenz curve, an absolute fair line y = x was drawn. The area between the curve and the absolute fair line is denoted by A. The area below the actual Lorenz curve is B. As A/(A + B) reflects the degree of inequitable distribution, the calculated value is known as the Gini coefficient (41).

The Gini coefficient ranges from 0 to 1. A smaller coefficient (closer to 0) indicates a more equitable distribution of resources, and a coefficient closer to 1 indicates a more concentrated resource entry. Gini coefficient < 0.2 indicates absolute average; values 0.2 < 0.3 indicate “relatively average;” values 0.3 < 0.4 indicate “reasonable;” values 0.4 < 0.5 indicates “large gap;” values 0.5 and above indicates “wide gap.” According to the United Nations Development Programme, 0.4 is the threshold of equitable and inequitable resource allocation (42). This study used a direct method to calculate the Gini coefficient. The equation can be written as follows:


$$\begin{array}{l} G=\left(-1\right)+\overset{n}{\underset{j=1}{\sum }}\frac{W_{j}}{W}\times \frac{Y_{j}}{Y}+2\overset{n-1}{\underset{j=1}{\sum }}\frac{W_{j}}{W}\times \left(1-V_{j}\right) \end{array}$$

where *G* represents the Gini coefficient, *Y*_*j*_ is the number of health resources per county, *Y* is the number of health resources in the province, *W*_*j*_ is the population in each county, *W*is the total population of the province, and *V*_*j*_ is the cumulative share of ${}^{Y_{j}}{/}_{Y}$ from *j* = 1 to *j* after sorting by the number of health resources per capita.

## 3. Results

### 3.1. Description of population health

#### 3.1.1. Change in population health

**Figure 1:**
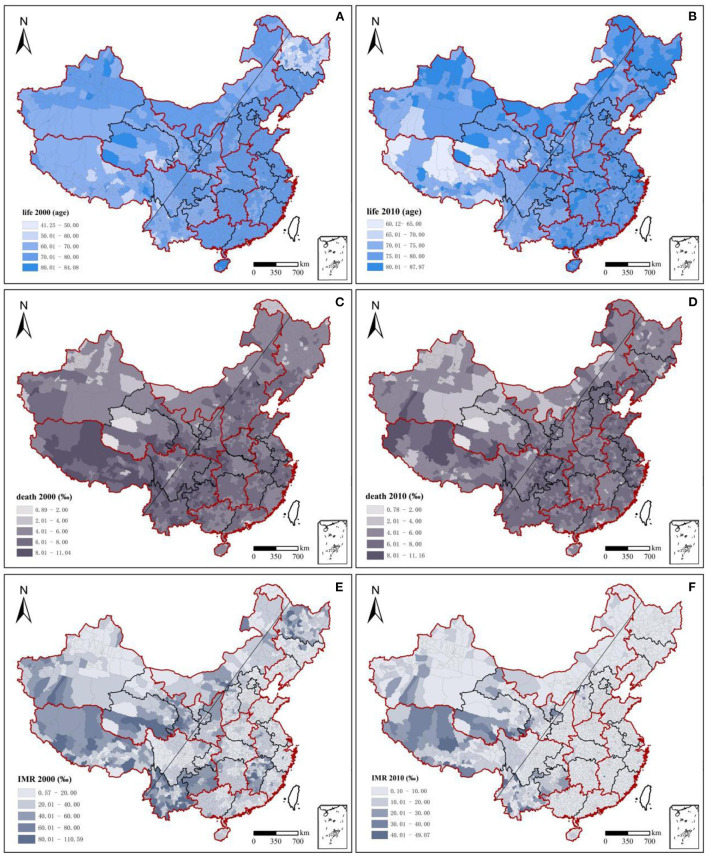
Changes and spatiotemporal evolution of population health, 2000–2010. Red outline: seven geographical regions' dividing lines; seven geographical regions: northeast China, east China, north China, central China, south China, southwest China, and northwest China. It was used to assist with east-west regional analysis; black outline: dividing lines of 31 provinces; slanting straight line: Hu Huanyong Line; Taiwan, Hong Kong, and Macao were not included in the study.

The overall population health in China increased considerably from 2000 to 2010 (Figure 1). Among the three health indicators, the average life expectancy at the county level increased from 71.40 years in 2000 (min. 41.25, max. 84.08) to 74.80 years in 2010 (60.12, 87.97), with an average annual growth rate of 0.52% (Table 2, Figures 1A, B). The infant mortality rate showed an obvious decreasing trend, from 28.40% in 2000 (0.89, 11.04%) to 13.10% in 2010 (0.78, 11.16%), with a decrease of 53.87% (Table 2, Figures 1C, D). The total mortality rate decreased from 32.20 in 2000 (0.57, 110.59%) to 13.10% in 2010 (0.10, 49.07%), with a decrease of 59.32% (Table 2, Figures 1D, E). Population health in the western region was lower than that in the eastern region, but the increase in population health in the western region was more significant. In which the average life expectancy in southwest China was 75.35 years in 2010, with an average annual growth rate of 0.97% since 2000; this was significantly higher than the national average of 0.52%. In 2010, the death rate and infant mortality rate were 6.55 and 11.17%, with average annual decline rates of 1.09 and 13.52%, respectively. Life expectancy, death rate, and infant mortality rate in northwest China were 71.23 years, 5.76, and 40.80% in 2000, respectively, with a total change of 7.86, −6.30, and −77.11% in 10 years.

#### 3.1.2. Spatiotemporal distribution characteristics of population health

**Figure 2:**
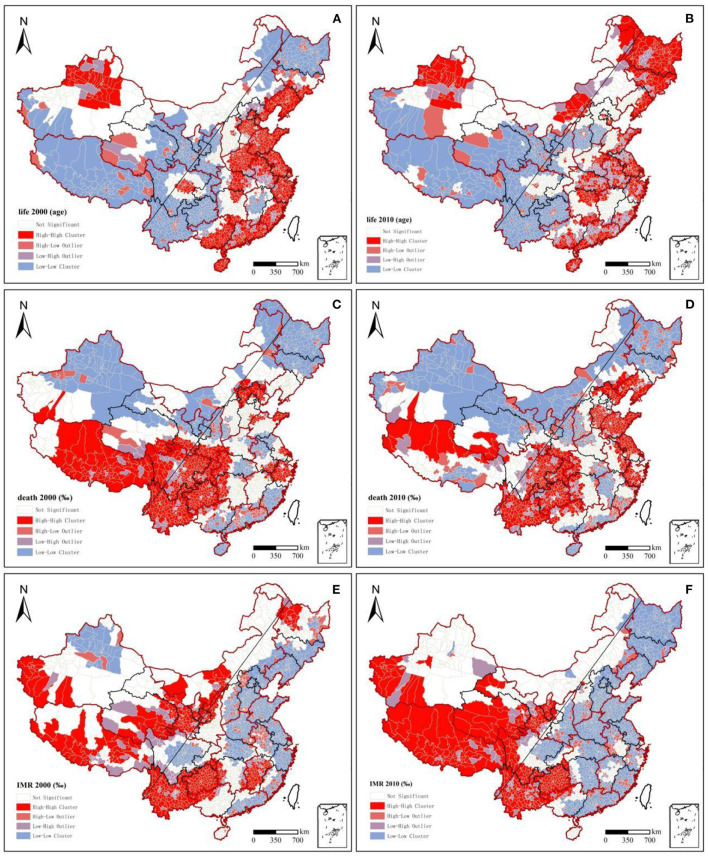
Lisa clustering charts of population health, 2000–2010.

National population health in 2000 and 2010 demonstrated significant spatial autocorrelation, and the spatial clustering patterns were relatively fixed (Figures 2A–D). Moran's *I* indices of the three health indicators were all >0 and passed significance tests at the 95% level. Moran's *I* indices of life expectancy, death rate, and infant mortality rate decreased from 0.313, 0.229, and 0.254 in 2000 to 0.200, 0.204, and 0.168 in 2010, respectively. This means that the degree of spatial clustering of population health in the county declined. Over time, the population's health status gradually improved in each region.

From 2000 to 2010, the distribution pattern of national population health became more stable, and population health showed a decreasing trend from the northwest and the east to the southwest. High-high clusters of life expectancy and low-low clusters of death rate and infant mortality rate were stably distributed in the eastern coastal region. In contrast, the low-low clusters of life expectancy and mortality and infant mortality were increasingly regionally concentrated during the study period, mainly in southwest China. Regarding life expectancy in 2000, high-high clusters were mainly concentrated along the eastern coast, but a low-low cluster area appeared in Jiangxi Province. Low-low clusters were generally found in the northeast and southwest China, whereas high-high cluster areas appeared in the Sichuan Basin and around the Tianshan Mountains in Xinjiang. In 2010, the eastern coastal area remained a high-high cluster area, while northeast China became a high-value area, and the low-value area in Jiangxi shrunk. The original high-high clusters in Beijing, Tianjin, and Hebei in 2000 became a high-value aggregation only in Beijing and Tianjin. The aggregation characteristics of the infant mortality rate and life expectancy from 2000 to 2010 were extremely similar, but with a converse pattern. Low-low clusters of infant mortality around Tianshan in Xinjiang in 2000 vanished. For the low-low clusters in southwest China, the range of high values continued to expand from south to north, and the degree of aggregation increased. Regarding the death rate, north China and southern coastal areas were mainly low-low clusters and remained stable across both years. Tibet and southwest China, including Yunnan, Guizhou, Sichuan, and Chongqing, as well as their surrounding areas, had stable high-high clusters. A significant change from low-low clusters in 2000 in Shandong Province to high-high clusters in 2010 was found. The Beijing-Tianjin agglomerations and Yangtze River Delta agglomerations, though surrounded by high-high clusters, had low-low or non-significant clusters.

### 3.2. Description of health resources

#### 3.2.1. Change in health resources

**Figure 3:**
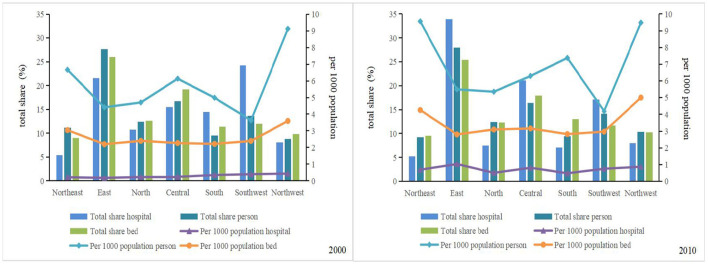
Regional changes in health resources, 2000–2010. Total share hospital means the number of hospitals in a region as a percentage of the national total; Per 1,000 population hospital means the number of hospitals for every 1,000 persons. Others are the same (below).

The overall health resource allocation per 1,000 persons in China showed continuous growth from 2000 to 2010 (Figure 3, Table 2). The number of hospital beds per 1,000 persons increased from 2.38 in 2000 (0.41, 7.99) to 3.63 in 2010 (0.33, 12.90), with an overall growth rate of 49.58% and an average annual growth rate of 4.58% (Table 2). Northwest China led the country in terms of the number of hospital beds per 1,000 persons, with a growth rate of 28.76% from 2000 to 2010. Meanwhile, east China and south China had the lowest amounts of health bed allocations, increasing from 2.20 and 2.22 in 2000 to 2.80 and 2.82 in 2010, respectively (Figure 3). The number of health technicians per 1,000 persons increased from 3.63 in 2000 (0.44, 24.78) to 4.37 in 2010 (0.51, 31.86), with an overall growth rate of 20.39% and an average annual growth rate of 2.08% (Table 2). Health staffing showed a clear inequality between the regions. For example, health technicians per 1,000 persons in the northeast and northwest China rose from 6.66 and 9.56 in 2000 to 9.56 and 9.48 in 2010, respectively. South China accounted for only 39.77 and 44.13% of the values in northwest China in 2000 and 2010, respectively. From 2000 to 2010, the government strongly encouraged the development of private medical institutions owing to a large number of private hospitals being set up. The values of health facilities per 1,000 persons rose rapidly in all regions, from 0.29 in 2000 (0.01, 2.91) to 0.74 in 2010 (0.01, 8.97), with an overall growth rate of 155.17% and an average annual growth rate of 9.82% (Table 2). The number of health facilities per 1,000 persons in east China increased from a low of 0.18 (in 2000) to a high of 1.04 (in 2010). Meanwhile, the values in northwest China–0.44 in 2000 and 0.87 in 2010—were significantly higher than the national regional averages of 0.29 and 0.74, respectively.

Figure 3 demonstrates the total share of health resources by region. The total ratio of health resource allocation in the western region was ~2.91 and 3.19 times higher than that in the eastern region in 2000 and 2010, respectively. However, northwest China reported lower percentages of the national total for health resources over the past decade. The proportions of health facilities, health technicians, and hospital beds in the national total ranged from 8.13, 8.78, and 9.82% in 2000 to 8.03, 10.38, and 10.25% in 2010, respectively. The eastern coastal region has increasingly contributed to China's total health resource allocation. Among them, the proportion of health resources in east China to the national total was maintained at ~25–30%, this accounted for the highest proportion of medical and health resources in the country. South China, southwest China, and northeast China showed significant downward trends. The decreases (2010–2000) in the total share were 16.77.13.98, and 5.09%, respectively.

#### 3.2.2. Spatial inequality in health resources

**Figure 4:**
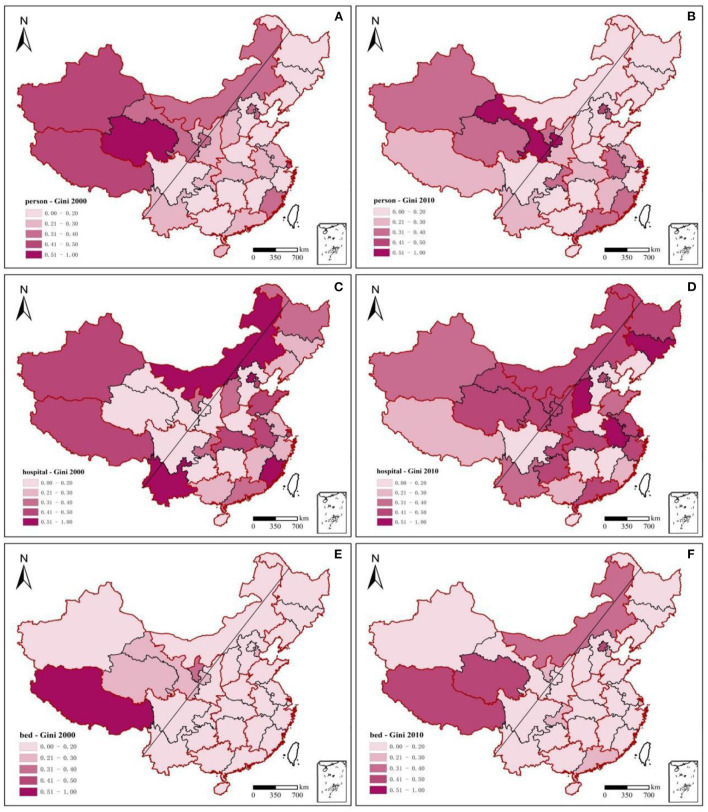
Gini coefficients of health resources across 31 provinces, 2000–2010.

This study used the Gini coefficient to measure the spatial inequality of health resources. China's health resource allocation was relatively equal between 2000 and 2010. In particular, inequality in the allocation of health institutions has decreased, while inequality in hospital beds and health technicians has increased significantly. The Gini coefficient of health facilities decreased from 0.1418 (in 2000) to 0.0681 (in 2010). The Gini coefficient of hospital beds increased from 0.0232 (in 2000) to 0.0309 (in 2010), whereas that of health technicians increased from 0.0002 (in 2000) to 0.0244 (in 2010). Using the “Hu Huanyong Line” (43) as the boundary, the inequality in medical resource allocation in the western region was significantly higher than that in the east in 2000. In 2010, the inequality between the east and west strengthened (Figure 4). Spatial differentiation of health technicians in 2010 was slightly lower than that in 2000, and the Gini coefficient of health technicians in northwest China was significantly higher than that of other regions. Among these, the Gini coefficients of Qinghai (0.54) in 2000 and Shanghai (0.74) and Gansu Province (0.50) in 2010 were higher than the alert value of 0.5, with obvious inequalities in health staffing. The Gini coefficient of health facility allocation fluctuated significantly in 2010 compared to that in 2000, and the degree of inequality deepened significantly. The degree of inequality in the eastern region increased from inland to coastal areas. At the same time, the degree of inequality in the western region decreased but was still significantly higher than that in the eastern region. From 2000 to 2010, the levels of inequality in northwest and north China remained high, and the areas were always on alert. The inequality from west to east in northwest China and from north to south in north China further increased in 2010 compared to that in 2000. The differences in the regional allocations of hospital beds from 2000 to 2010 were relatively equal. However, the Gini coefficients of hospital bed allocation in Tibet (0.53) and Qinghai (0.46) in 2000 and in Tibet (0.42) and Beijing (0.42) in 2010 were much higher than those in other regions. These findings suggest that the differences in the regional allocation of health resources in the western region (especially in the northwestern region) and eastern urban agglomerations were relatively disparate from 2000 to 2010.

### 3.3. Health resources influencing population health

OLS test should be performed before conducting MGWR regression in general. OLS coefficients was overall significant (Tables 3, 4). The maximum value of the variance inflation factor (VIF) was 4.97, below the alert value of 10. It indicated no serious covariance problems. Meanwhile, for the data normality test, the deviated values of all variables were consistent with the hypothesis of a normal distribution in terms of the degree and direction of deviation.

**Table 3:** OLS regression results; *p*-value (*p* < 0.10).

**Variable names**	**2000**			**2010**		
	**Coef**	***P***	**VIF**	**Coef**	***P***	**VIF**
**Life expectancy**						
Minimum temperature	0.082	0.000	1.180	−0.047	0.000	1.283
Housing floor area per capita	0.135	0.000	1.310	0.058	0.000	1.356
Urban and rural residents savings deposit balance	0.000	0.000	1.211	0.000	0.001	1.225
Urbanization	0.000	0.000	1.001	0.072	0.001	1.164
Illiterate population aged 15 years and above	−0.000	0.000	1.058	−0.000	0.002	1.074
Normalized difference vegetation index	−4.222	0.000	1.167	1.563	0.001	1.124
GDP per capita	0.000	0.074	3.925	−0.000	0.090	3.212
Health facilities per 1,000 persons	−0.592	0.070	4.972	−0.072	0.018	1.512
Health technicians per 1,000 persons	0.042	0.047	4.311	0.021	0.026	2.000
Hospital beds per 1,000 persons	0.001	0.099	4.510	−0.011	0.082	3.168
**Death rate**						
Minimum temperature	0.017	0.000	1.180	−0.003	0.043	1.283
Housing floor area per capita	0.014	0.002	1.310	0.038	0.000	1.356
Urban and rural residents savings deposit balance	−0.000	0.000	1.211	−0.000	0.025	1.225
Urbanization	−0.000	0.072	1.001	−0.014	0.001	1.164
Illiterate population aged 15 years and above	0.000	0.000	1.058	0.000	0.001	1.074
Normalized difference vegetation index	1.208	0.000	1.167	2.018	0.000	1.124
GDP per capita	−0.000	0.027	3.925	0.000	0.046	3.212
Health facilities per 1,000 persons	0.251	0.001	4.972	−0.009	0.028	1.512
Health technicians per 1,000 persons	−0.014	0.002	4.311	−0.004	0.064	2.000
Hospital beds per 1,000 persons	0.001	0.096	4.509	−0.002	0.091	3.168
**Infant mortality rate**						
Minimum temperature	0.456	0.000	1.180	0.184	0.000	1.283
Housing floor area per capita	−1.008	0.000	1.310	−0.197	0.000	1.356
Urban and rural residents savings deposit balance	−0.000	0.000	1.211	−0.000	0.000	1.225
Urbanization	−0.000	0.000	1.001	−0.052	0.001	1.164
Illiterate population aged 15 years and above	0.000	0.081	1.058	−0.000	−0.061	1.074
Normalized difference vegetation index	5.792	0.054	1.167	−8.308	0.000	1.124
GDP per capita	−0.001	0.035	3.925	−0.000	0.015	3.212
Health facilities per 1,000 persons	1.633	0.095	4.972	0.077	0.014	1.512
Health technicians per 1,000 persons	−0.144	0.020	4.311	−0.027	0.058	2.000
Hospital beds per 1,000 persons	0.173	0.044	4.509	0.212	0.006	3.168

**Table 4:** OLS regression results (ratio); *p*-value (*p* < 0.10).

**Variable names**	**Coefficient**	**P**	**VIF**
**Life expectancy (ratio)**			
Minimum temperature	0.000	0.000	1.000
Housing floor area per capita	0.003	0.068	1.031
Urban and rural residents savings deposit balance	0.000	0.044	1.424
Urbanization	−0.002	0.056	1.080
Illiterate population aged 15 years and above	0.006	0.003	1.012
Normalized difference vegetation index	0.027	0.004	1.011
GDP per capita	−0.000	0.021	1.601
Health facilities per 1,000 persons	−0.000	0.000	1.005
Health technicians per 1,000 persons	0.000	0.026	1.065
Hospital beds per 1,000 persons	0.001	0.051	1.577
**Death rate (ratio)**			
Minimum temperature	0.000	0.000	1.000
Housing floor area per capita	−0.054	0.082	1.031
Urban and rural residents savings deposit balance	0.002	0.006	1.424
Urbanization	0.011	0.015	1.080
Illiterate population aged 15 years and above	0.008	0.012	1.013
Normalized difference vegetation index	0.014	0.063	1.011
GDP per capita	0.000	0.006	1.601
Health facilities per 1,000 persons	0.000	0.017	1.005
Health technicians per 1,000 persons	−0.002	0.060	1.065
Hospital beds per 1,000 persons	0.012	0.057	1.577
**Infant mortality rate (ratio)**		
Minimum temperature	−0.000	0.000	1.000
Housing floor area per capita	−0.038	0.024	1.031
Urban and rural residents savings deposit balance	0.000	0.039	1.424
Urbanization	0.000	0.099	1.080
Illiterate population aged 15 years and above	0.007	0.018	1.013
Normalized difference vegetation index	0.036	0.073	1.011
GDP per capita	0.000	0.019	1.601
Health facilities per 1,000 persons	0.000	0.081	1.005
Health technicians per 1,000 persons	0.004	0.033	1.065
Hospital beds per 1,000 persons	−0.031	0.000	1.577

Ratio = 2,010/2,000 values.

After the variables were differentiated by the first order, augmented dickey-fuller test (ADF) unit root test was used, the variables showed a steady state, and no unit root existed. Adjusted *R*-square of MGWR was >0.57 (Table 5). The AICc values and the sum of the squared residuals were lower than those of the GWR. This indicated a higher MGWR's robustness and regression results' credibility. When *p* < 0.05, the regression coefficient of health technicians per 1,000 persons on population health was significant in 2000 and 2010 (44) (Table 6).

**Table 5:** *R*^2^ and Adjusted *R*^2^ of MGWR regressions.

**Variable**	***R^2^***	**2000 *R^2^***	**2010 *R^2^***
Life		0.75	0.71
Death		0.72	0.67
IMR		0.63	0.57
Life (ratio)	0.72		
Death (ratio)	0.67		
IMR (ratio)	0.62		

**Table 6:** MGWR regression results; *p*-value (*p* < 0.05).

**Variable**	**2000**			**2010**		
	**Life**	**Death**	**Imr**	**Life**	**Death**	**IMR**
TEMP	0.000	0.000	0.000	0.000	0.279	0.000
Housing	0.000	0.000	0.000	0.000	0.000	0.000
Saving	0.000	0.000	0.000	0.000	0.138	0.000
Urban	0.289	0.985	0.283	0.000	0.000	0.000
Unedu	0.000	0.000	0.112	0.000	0.001	0.562
NDVI	0.000	0.000	0.046	0.000	0.000	0.000
GDP	0.622	0.045	0.391	0.862	0.465	0.104
Hospital	0.001	0.000	0.077	0.016	0.457	0.255
Person	0.000	0.000	0.003	0.000	0.038	0.020
Bed	0.984	0.923	0.398	0.651	0.855	0.000

In Tables 7, 8, MGWR directly reflects variables' impact scales. Different variables with significant regression coefficients were found to have very different impact scales. It is represented by the bandwidth. The bandwidth refers to the number of surrounding points for the estimated point in the regression. Different bandwidths for different variables are equivalent to different scales—some close to the global scale, and some, the local scale. Among them, the global scale represents that the number of surrounding points is infinitely close to the sample size. It indicates no spatial heterogeneity. Based on the degree of the factors' influence, it was gathered that the influence degree of health technicians per 1,000 persons (2010) on death rate was 2,857, which was a global scale. This indicated that there was no spatial heterogeneity. In other words, death rate for the above variable was essentially the same across regions in the corresponding years. In 2000, the impact scales of health technicians per 1,000 persons on life expectancy, death rate, and infant mortality rate in 2000 were 273, 65, and 298, respectively. This indicates that population health varied more spatially with changes in the number of health technicians. Once the impact scale was exceeded, the coefficients changed considerably. The impact of health technicians per 1,000 persons on the death and infant mortality rates increased from 56 and 298 in 2,000–2,857 and 2,835 in 2010, respectively. This indicates that the impact of human resources on health tended to be consistent across regions from 2000 to 2010, and its impact has been continuously and steadily expanding.

**Table 7:** Statistical description of MGWR coefficients, 2000.

**Variable**	**Global scale**	**Coef**	**Bandwidth**	**Mean**	**STD**	**Min**	**Median**	**Max**
**Var. life**								
TEMP		0.153	673	0.361	0.417	−0.429	0.339	0.897
Housing		0.174	704	0.025	0.078	−0.257	0.041	0.152
Saving		0.248	247	0.102	0.142	−0.279	0.060	0.536
Unedu	Y	−0.065	2,857	−0.105	0.003	0.110	−0.106	−0.097
NDVI		−0.138	107	−0.219	0.281	−1.385	−0.176	0.286
Hospital	Y	−0.126	2,857	−0.013	0.001	−0.014	−0.013	−0.007
Person		0.158	273	0.219	0.411	−0.245	0.087	2.138
**Var. death**								
TEMP		0.124	1,812	−0.340	0.144	−0.502	0.384	−0.109
Housing		0.070	157	0.132	0.172	−0.305	0.126	0.531
Saving		−0.158	319	−0.113	0.127	0.440	−0.074	0.076
Unedu	Y	0.163	2,857	0.073	0.002	0.070	0.073	0.076
NDVI		0.150	802	0.163	0.080	0.027	0.161	0.320
GDP		−0.069	271	−0.071	0.154	−0.535	−0.031	0.210
Hospital		0.203	416	−0.184	0.375	−0.770	−0.097	0.502
Person		−0.207	65	0.251	0.943	−3.942	0.155	3.660
**Var. IMR**								
TEMP	Y	0.168	2,857	0.065	0.001	0.059	0.065	0.067
Housing		−0.257	49	−0.099	0.298	−1.867	−0.088	1.142
Saving		−0.197	1,782	0.057	0.048	−0.176	−0.038	−0.001
NDVI	Y	0.037	2,846	0.099	0.007	0.071	0.102	0.103
Person		−0.108	298	−0.246	0.483	−3.081	−0.135	0.258

All the above variables were statistically significant (p < 0.05); Y indicates that the variables belong to the global scale; “Var. life” indicates “Variable: life expectancy,” “Var. death” indicates “Variable: death rate,” “Var. IMR” indicates “Variable: infant mortality rate;” the same to Table 8.

**Table 8:** Statistical description of MGWR coefficients, 2010.

**Variable**	**Global scale**	**Coef**	**Bandwidth**	**Mean**	**STD**	**Min**	**Median**	**Max**
**Var. life**								
TEMP	Y	−0.115	2,857	0.089	0.002	0.084	0.089	0.092
Housing		0.114	70	−0.040	0.253	−0.866	−0.029	1.414
Saving		0.095	153	0.105	0.263	−0.302	0.017	1.517
Urban		0.479	45	0.525	0.314	−0.612	0.495	2.472
Unedu	Y	−0.060	2,857	−0.012	0.001	−0.013	−0.012	0.008
NDVI		0.065	698	−0.078	0.061	−0.163	−0.098	0.137
Person		0.091	354	0.083	0.195	−0.192	0.043	0.994
Hospital		−0.046	541	0.026	0.169	−0.371	−0.016	0.412
**Var. death**								
Housing		0.204	45	0.217	0.428	−2.320	0.221	1.115
Urban		−0.251	47	−0.307	0.346	−2.025	−0.297	1.134
Unedu		0.059	43	0.122	0.668	−2.125	0.083	2.514
NDVI		0.229	2,535	0.223	0.012	0.180	0.227	0.235
Person	Y	−0.049	2,857	0.000	0.003	−0.008	0.001	0.003
**Var. IMR**								
TEMP	Y	0.223	2,857	0.014	0.004	0.010	0.013	0.030
Housing		−0.193	51	−0.079	0.260	−2.073	−0.012	1.167
Saving		−0.105	2,096	−0.034	0.039	−0.167	−0.013	−0.004
Urban		−0.174	45	−0.105	0.407	−2.528	−0.054	3.969
NDVI	Y	−0.173	2,847	−0.040	0.008	−0.070	−0.037	−0.033
Person	Y	−0.057	2,835	0.013	0.009	−0.015	0.017	0.020
Bed		0.117	395	0.050	0.074	−0.152	0.042	0.408

**Figure 5:**
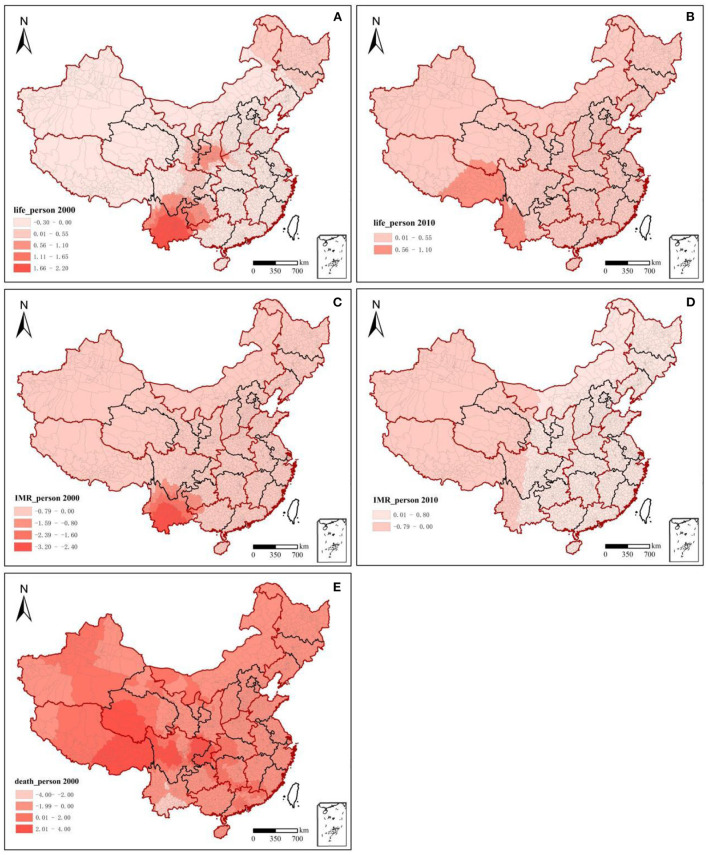
Spatial patterns of health technicians influencing population health, 2000–2010. Beta coefficients (*p* < 0.05); white blank: values were not statistically significant; Taiwan, Hong Kong, and Macao were excluded from the study.

**Figure 6:**
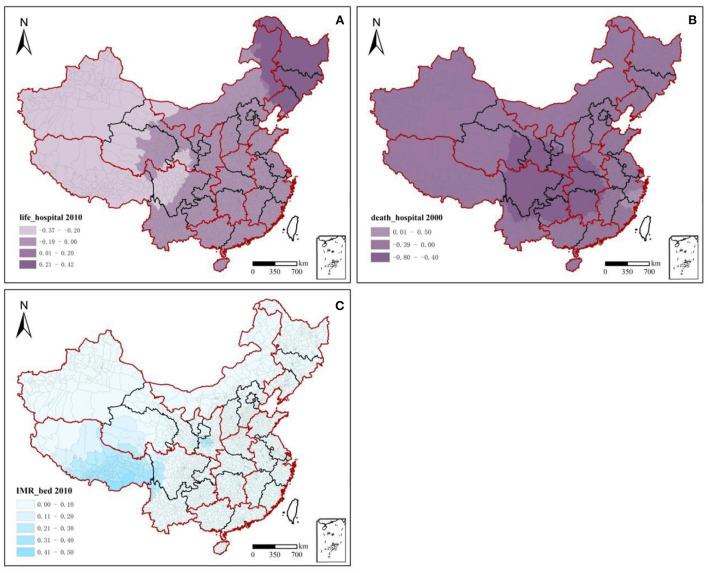
Spatial patterns of health facilities and hospital beds influencing population health, 2000–2010.

The spatial influence patterns of significant independent variables on dependent variables are presented in Figures 5, 6. The statistical description of each MGWR coefficient is presented in Tables 7, 8. The number of health technicians per 1,000 persons had a significant positive effect on life expectancy in 2000 and 2010. The coefficients indicated that for each unit increase in health technicians per 1,000 persons, life expectancy increased by 0.158–0.091 years, with a mean increase of 0.219 and 0.083 years, respectively. From the absolute values of the coefficients, it was gathered that the degree of influence was at the forefront of all variables, with a significant effect on the increase in life expectancy.

Health technicians per 1,000 persons significantly and negatively affected death and infant mortality rates. Each unit of health technicians per 1,000 persons increased between 2000 and 2010, and the death rate decreased by 0.207–0.049%, with a mean decrease of 0.251–0.000%. Regional differences in the rate of decrease were obvious, but the values tended to decrease. Meanwhile, the infant mortality rate decreased by 0.108–0.057%, with a mean decrease of 0.246–0.013%, with little regional difference in the rate of decrease. In terms of time changes, and due to rapid socioeconomic development, the causes affecting population health have become increasingly complex. From 2000 to 2010, the degree of influence of health technicians on life expectancy, death rate, and infant mortality rate weakened with decreases of 42.14, 76.33, and 47.22%, respectively. In terms of spatial variation, the high-value impact areas of health technicians on population health were relatively concentrated. The impact of high-value areas of health technicians per 1,000 persons on life expectancy decreased from the eastern part of northwest China and the southeastern part of southwest China (2000) to the southern part of southwest China (2010). From 2000 to 2010, the differences in the degree of influence of health technicians per 1,000 persons on the death rate and infant mortality rate between the eastern and western regions were not significant; the intensity of these effects tended to weaken. The high-impact values were mainly concentrated in the western region, especially in southwest China. Among them, the effect of health technicians per 1,000 persons on the death rate in southwest and northwest China shifted from a positive one in 2000 to a negative one in 2010. Meanwhile, the negative influence of health technicians per 1,000 persons on the infant mortality rate in the eastern region was significantly reduced. This indicates that the inputs of health technicians significantly reduced the death rate in the west. However, the utility of reducing the infant mortality rate in the east showed a diminishing marginal efficiency.

### 3.4. The impact on health change

**Figure 7:**
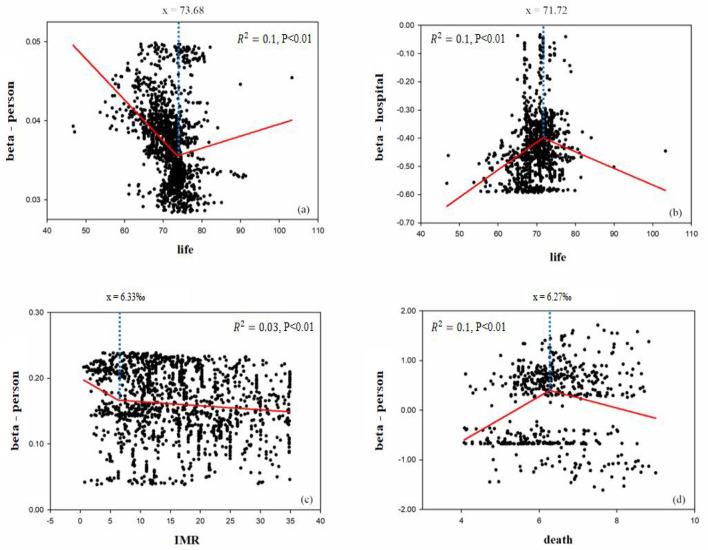
Impact on population health of health resource enhancement, 2000–2010. Beta coefficients (*p* < 0.05); y = beta coefficient (in 2010)/beta coefficient (in 2000) for Y-axis; X-axis was life expectancy data (in 2000).

The significant beta coefficient ratio (2010/2000) of health resources and the corresponding population health values in 2000 are curve-fitted in Figure 7. This study analyzed the effects of health resource enhancement on population health from 2000 to 2010. The results showed that the marginal impact utility of health technicians per 1,000 persons on life expectancy was V-shaped, with the boundary of life expectancy being 73.68 years. The marginal impact utility of health technicians for life expectancy per 1,000 persons decreased rapidly from 55.00 to 73.68 years, whereas the marginal impact of health technicians per 1,000 persons increased slowly when life expectancy was above 73.68 years. This indicates that when life expectancy was below 73.68 years, the gains in life expectancy from health manpower inputs tended to decrease rapidly when life expectancy was below 73.68 years, whereas gains in life expectancy tended to increase slowly. The marginal effect utility of health facilities per 1,000 persons on life expectancy was bounded by 71.72 years and had an inverted V-distribution. The values show that when life expectancy is below 71.72 years, health facilities should be built and invested in more, and when life expectancy is above 71.72 years, the function of health facilities becomes less important. The marginal effect of health technicians per 1,000 persons on the death rate had an inverted V-shaped distribution with a threshold of 6.27%. The marginal effect utility of health technicians per 1,000 persons on the infant mortality rate was “oblique L-shaped,” which was slowly decreasing. Among them, when the infant mortality rate was below 6.33%, the marginal impact utility of health technicians per 1,000 persons on the infant mortality rate was significantly greater than that when the infant mortality rate was above 6.33. This indicates that when infant mortality rates range from 0.00 to 6.33%, investments in human resources for health should be increased.

## 4. Discussion

Health resources are immaterial labor products, and their production synchronizes with consumption. That is, they occur simultaneously, are inseparable, and are identical in time and space (45). However, health service system is limited by geographic and spatial factors. Owing to policies such as the *Health Poverty Reduction* (46), the total and per 1,000 persons of health resource allocations in the west were much higher than those in the east. Health resource allocation in east China is influenced by the regional economy, urbanization, and population migration flows (47). The ratio of health resources per 1,000 persons was relatively low, with an average annual growth rate of only 2.08%. Among them, the ratio of total health resources in northeast China showed a flat downward trend. The main reason for this is the heavy industry decline and industrial restructuring (48). This has led to a low fertility rate and high emigration rate in terms of demographic characteristics (49), indirectly affecting health resource allocation. It indicates that regional health resources are set mainly according to economic development, administrative affiliation, and policy-oriented settings in current. The unreasonable segmentation and duplication allocations led to the coexistence of under-investment, waste of health resources, and large differences in spatial layout. In 2016, *the Tripartite Five Report* proposed that effective health system planning should be based on demographic structure, epidemiology, and urban development planning from a long-term perspective for the development of inter-regional differentiated health resource input policies (50). Therefore, based on geographic information system, the government should visualize the relationship between health resources and future population health needs. Subsequently, visualization operations and spatial statistical operations are performed to improve the scientific and rational nature of the regional planning of health resources.

Health resource allocation is not essentially a solution to the per 1,000 persons ownership problem, but rather a process of rationalizing resource layout, considering the geographic situation, economy, and population health status in each region (51). At present, the spatial allocation of health resources nationwide is measured by population equity and is allocated based on per 1,000 persons health resource ownership. Although this behavior has achieved a more efficient use of health resources, it goes against the equalizing ideas of basic public services. More high-quality health resources have shifted to the large, dense populations of eastern urban agglomerations. However, high-density population concentrations (52) and limited health resources (53) have inevitably led to high inequities in health resource allocation in cities such as Beijing and Shanghai. In the sparsely populated western region, the total amount of resource allocation was not low because of the policy tilt (54). However, the equality and accessibility of resource allocation were poor. Health service accessibility is the main factor affecting the spatial distribution of health resources in the western region (55). Economic development in the west lagged behind and transportation was relatively inconvenient. If health resources were allocated according to the population distribution, the health service radius would be too large. Residents would experience an increase in the distance and travel costs to medical appointments (56). Therefore, the government should develop a resource allocation plan that complements the actual situation in each region. This is based on the combined effects of economic conditions and geographic environment. In the eastern region, resource utilization efficiency and resources per 1,000 persons are key to resource allocation. In the western region, increased attention should be paid to geographic equity when planning health resource allocation. On the one hand, attention should be paid to health resources per 1,000 persons. On the other hand, we also consider the impact of resident population size and medical service radius on resource allocation.

Health staffing had increased, increasing from 449.10 million in 2000 to 587.62 million in 2010, with an annual growth rate of 30.84% and an average annual growth rate of 2.47% (57). However, it has not yet been able to meet the growing medical needs of people in eastern and western regions. From 2000 to 2010, as urbanization in the east increased, large urban agglomerations emerged and the urban population size continued to expand. This led to the development of health resources that failed to match population density. The growth rate of resources did not match that of the resident population. At the same time, there were siphoning effects of health resources in extremely economically developed regions (58), which inevitably led to the coexistence of waste and shortages in health human resources in the eastern region. The accessibility and availability of health services are poor in remote areas in the west because of economic backwardness and the area being vast and sparsely populated. There were a series of problems, such as the inability to attract and retain health technicians. The number of health institutions and beds in most areas was set up as standards, but medical equipment and health technicians could not be matched. This has resulted in poor access to medical services. Therefore, the government should adjust the spatial layout of health resources and promote the optimal integration of health manpower *via* health planning. Attention should be paid to factors such as economic status and the geographic environment. Targeted health staffing policies should be adopted based on the actual situation in each region. In the developed regions, the allocated number of health technicians remains unchanged. The education and title structures of human resources are optimized through the job management system, personnel training, and re-education (59, 60). In less-developed areas, the “*three supports and one assistance*” policy should be improved. Meanwhile, the government acts as a support assessment system to ensure the effectiveness of targeted health support. It attracts and retains talent through employment orientation, performance allocation, salary and benefits, title promotion, and other health-oriented policies (61). At the same time, social medical services are strongly encouraged to quickly replenish health technicians and address staff shortages. Between developed and less-developed regions, we should build a platform for sharing telemedicine technology and human resources through Internet big data, cloud platforms, mobile Internet, the Internet of Things, and other new technologies (62). Hospitals in the east and west need to establish fixed-point medical support. Through the web visualization platform, they can conduct a series of activities such as specialist clinics, specialist consultations and surgical procedure assistance. Thus, less developed regions can conveniently enjoy high-quality medical resources, effectively alleviating the unequal distribution of health technicians between regions.

Population health is influenced by many factors in concert, and health resources are not the only decisive factor affecting health. In this study, parameters including medical resources, economic education, and other factors could explain only 67.50% of the dependent variable. This indicates that population health is influenced by several factors (63). We found that housing floor area per capita and NDVI were the key factors influencing population health, as correction variables (Supplementary Figures 1–3). Moderate NDVI values could benefit population health (64). This was particularly pronounced in the east. The housing floor area per capita has a negative effect on population health (65). However, it had little effect on relatively backward and sparsely populated regions in 2000 and 2010. Therefore, against the backdrop of rapid urbanization, attention should be paid to improving the natural and social environments.

## 5. Conclusions

This study considered county population health and health resources as research objects. First, we analyzed the changing trends and regional differences in county population health in China from 2000 to 2010. Second, we explored the spatial distribution patterns and spatial clustering characteristics of health resource allocation across counties. Finally, we discussed the impact of health resource enhancement on health and its regional difference characteristics based on MGWR and curve fitting. In this study, we clarified the impact of health resource enhancement and its spatiotemporal relationship with population health. At the same time, we found a quantitative relationship exists between population health and health resource inputs.

The main findings are as follows:

(1) From 2000 to 2010, the overall health of the nation's residents continued to rise at a relatively stable rate. The health of the eastern population was better than that of the western population. However, the increase in population health in the west was obvious, and there was more room to improve the population health level. The distribution pattern of national population health was stable and consistent with the regional development status. The population health level showed a decreasing trend from the northwest and east, to the southwest.
(2) National health resource allocation improved from 2000 to 2010. The total and per 1,000 persons health resource allocations in the west were much higher than those in the east. East China is the country's main source of health resources. The inequality allocation of health institutions decreased, whereas inequalities in hospital beds and health technicians increased significantly. The unequal resource allocation in the west was higher than that in the east. Northwest China, first-tier cities, and coastal urban clusters in the east reported poor equity in health resource allocation.
(3) The influence of health resources on population health did not rely on the physical configuration of institutions but instead on the overall allocation of healthcare manpower. From 2000 to 2010, health technicians per 1,000 persons had a significant positive effect on the population health. Moreover, its impact was consistent and continuously increasing across regions.

A quantitative relationship existed between population health and health resource inputs from 2000 to 2010. The number of health technicians per 1,000 persons should be actively increased, when life expectancy ranged from 73.68 to 84.08 years, the death rate ranged from 6.27 to 9.00%, and the infant mortality rate ranged from 0.00 to 6.33%. The number of health facilities per 1,000 persons should be reasonably decreased when life expectancy ranges between 71.21 and 82.00 years.

## 6. Shortcomings and prospects

This study provides a reference for coordinated allocation and spatial planning of regional population health and health resources. However, owing to the limitations of many factors, this study has the following shortcomings, which call for improvement.

In this study, population health indicators, such as life expectancy, were a cumulative concept; that is, the impact of health resources on life expectancy was not immediate, but a result of long-term accumulation. Therefore, the impact of health resources on population health level possesses a certain lag. In future studies on their relationship, the lagged effects of health resources on resident health should be explored to make the conclusions more reliable.

Indicators for evaluating health resources and population health need to be more rigorous and scientific. In this study, based on the current situation of domestic and foreign research and the difficulty of data acquisition, health facilities per 1,000 persons, health technicians per 1,000 persons, and hospital beds per 1,000 persons were used as the indicators of health resources. However, the selection of health resource indicators often ignores the emphasis on quality and the individual differences in business ability. In terms of population health, the infant mortality rate is contingent on time. If the population base of an area is small, then the number of infants must be small and the death toll will change slightly, leading to a large fluctuation in the death rate. Thus, in sparsely populated areas, infant mortality rates are highly uncertain in terms of space and time.

## Data availability statement

The datasets presented in this article are not readily available because data acquisition requires contacting the corresponding author to obtain consent. Requests to access the datasets should be directed to wangli@igsnrr.ac.cn.

## Author contributions

LQ wrote the manuscript. LW revised it. LY and HL advised it. All authors contributed to the article and approved the submitted version.
