# Supplementary material for: The impact of health resource enhancement and its spatiotemporal relationship with population health
Source: Front Public Health. 2023 Jan 9;10:1043184. doi: 10.3389/fpubh.2022.1043184 (PMC9868711; doi:10.3389/fpubh.2022.1043184)
Supplement: Supplementary file 1 [file Data_Sheet_1.docx]

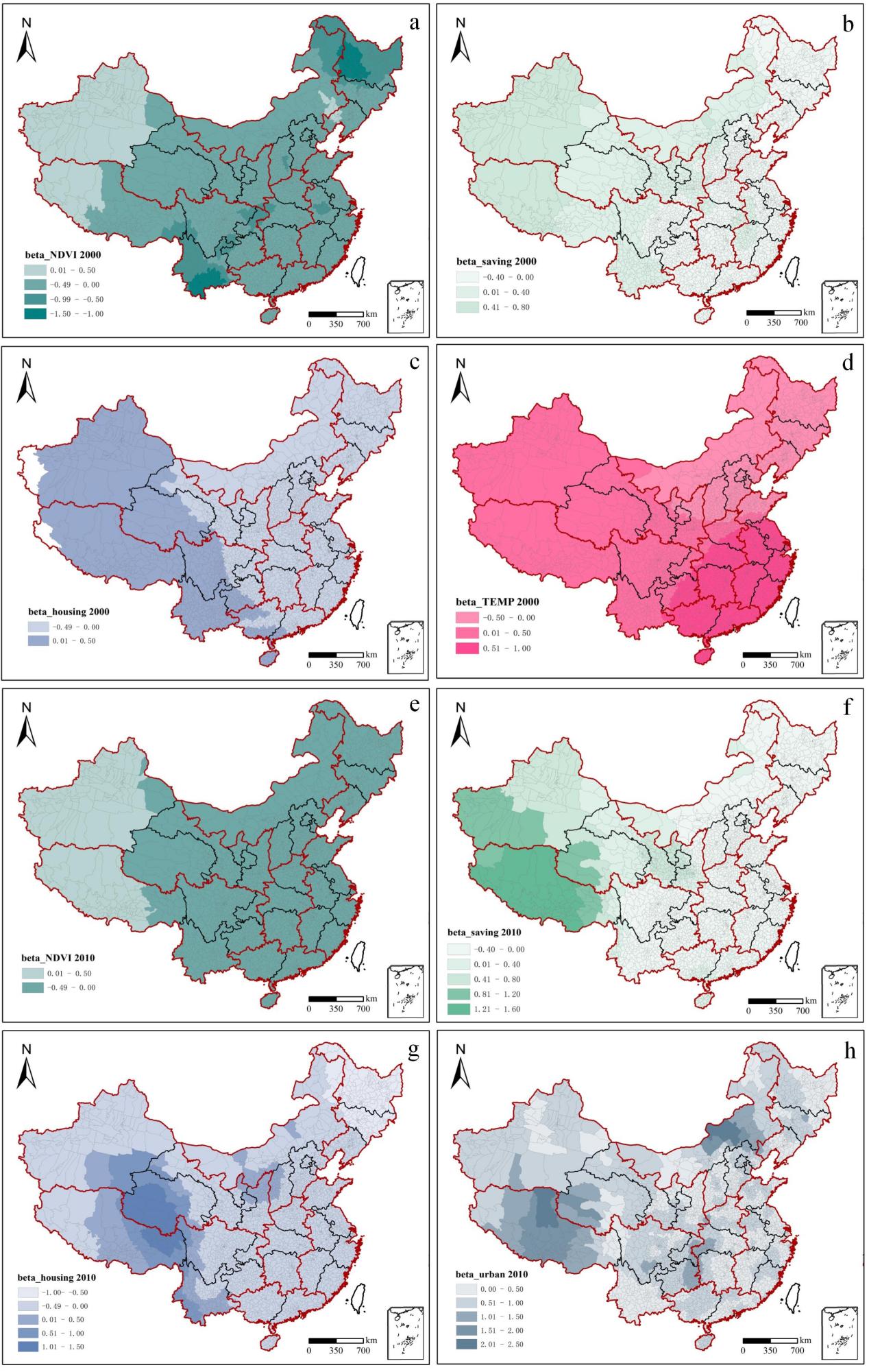


Figure S1. Spatial patterns of other factors influencing life expectancy, 2000–2010


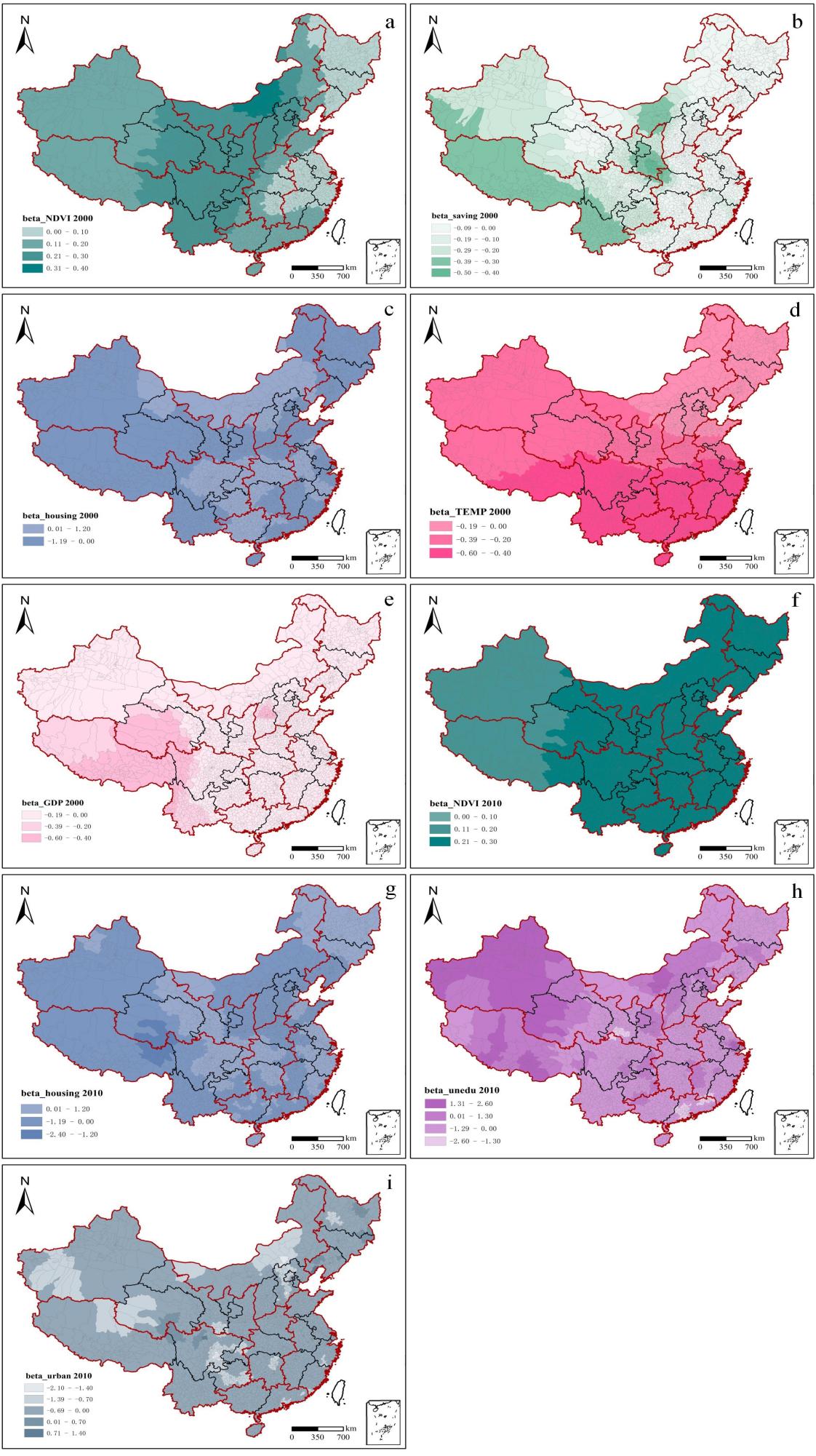


Figure S2. Spatial patterns of other factors influencing death rate, 2000–2010


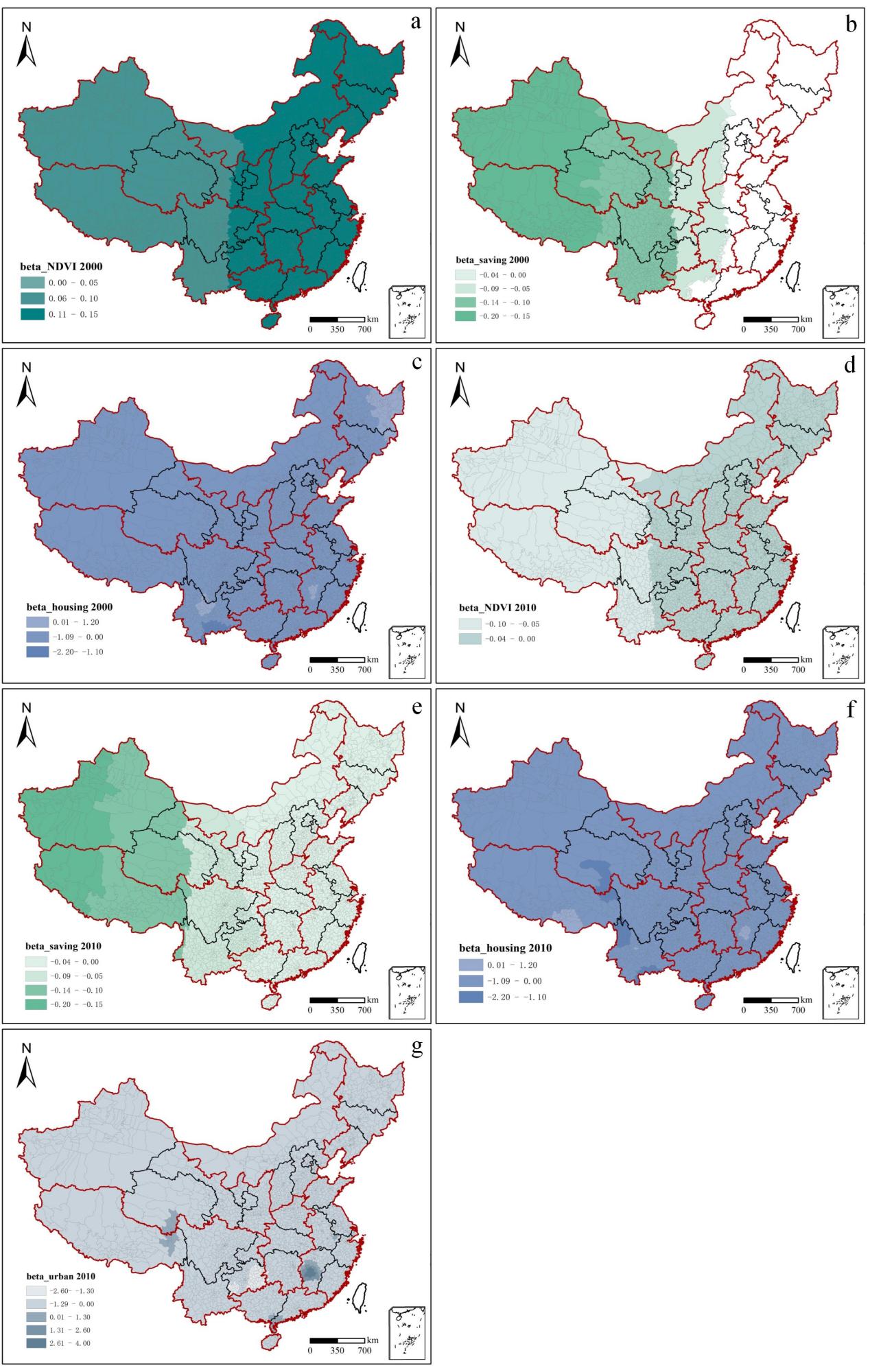


Figure S3. Spatial patterns of other factors influencing infant mortality rate, 2000–2010
